# Crystal structure of di­chlorido­bis­(dimethyl *N*-cyano­dithio­imino­carbonate)cobalt(II)

**DOI:** 10.1107/S2056989015023439

**Published:** 2016-01-01

**Authors:** Mouhamadou Birame Diop, Libasse Diop, Allen G. Oliver

**Affiliations:** a Laboratoire de Chimie Minérale et Analytique, Département de Chimie, Faculté des Sciences et Techniques, Université Cheikh Anta Diop, Dakar, Senegal; b Department of Chemistry and Biochemistry, University of Notre Dame, 246, Nieuwland, Science Hall, Notre Dame, IN 46557-5670, USA

**Keywords:** crystal structure, cobalt(II), dimethyl *N*-cyano­dithio­imino­carbonate, tetra­hedral configuration

## Abstract

The structure of the mononuclear title complex, [{(H_3_CS)_2_C=NC  N}_2_CoCl_2_], consists of a Co^II^ atom coordinated in a distorted tetra­hedral manner by two Cl^−^ ligands and the terminal *N* atoms of two dimethyl *N*-cyano­dithio­imino­carbonate ligands. The two organic ligands are almost coplanar, with a dihedral angle of 5.99 (6)° between their least-squares planes. The crystal packing features pairs of inversion-related complexes that are held together through C—H⋯Cl and C—H⋯S inter­actions and π–π stacking [centroid-to-centroid distance = 3.515 **(su?)** Å]. Additional C—H⋯Cl and C—H⋯S inter­actions, as well as Cl⋯S contacts < 3.6 Å, consolidate the crystal packing.

## Abstract 2

The title cobalt(II) complex consists of a CoCl_2_ moiety linked to the terminal N atoms of two dimethyl *N*-cyano­dithio­imino­carbonate mol­ecules which leads to an overall distorted tetra­hedral configuration around the Co^II^ atom. Weak C—H⋯Cl and C—H⋯S hydrogen-bonding inter­actions consolidate the packing of the structure.

## Chemical context   

Dimethyl *N*-cyano­dithio­imino­carbonate with its two N and two S atoms has four possible coordination sites and hence should present a high coordination ability. The behaviour of N and S atoms according to Pearson’s concept as hard and soft donors, respectively, may allow coordination to both hard and soft Lewis acids. Despite this coordination property, the ligand has scarcely been studied. Only one crystalline compound with dimethyl *N*-cyano­dithio­imino­carbonate as a ligand has been reported previously (Kojić-Prodić *et al.*, 1992 ▸). The structure of this latter compound contains polymeric [Cu^I^Cl]_*n*_ chains flanked by two *N*-coordinating ligands. Because of the scarcity of data on this ligand, we have initiated a study of the inter­actions between cobalt(II) chloride hexa­hydrate and dimethyl *N*-cyano­dithio­imino­carbonate which has yielded the title complex, [{(H_3_CS)_2_C=NC  N}_2_CoCl_2_].
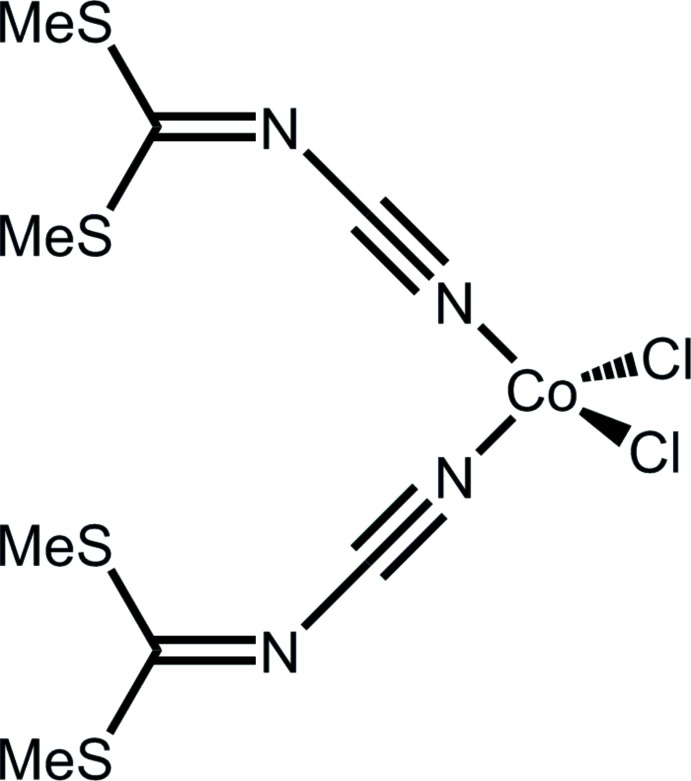


## Structural commentary   

**Figure 1:**
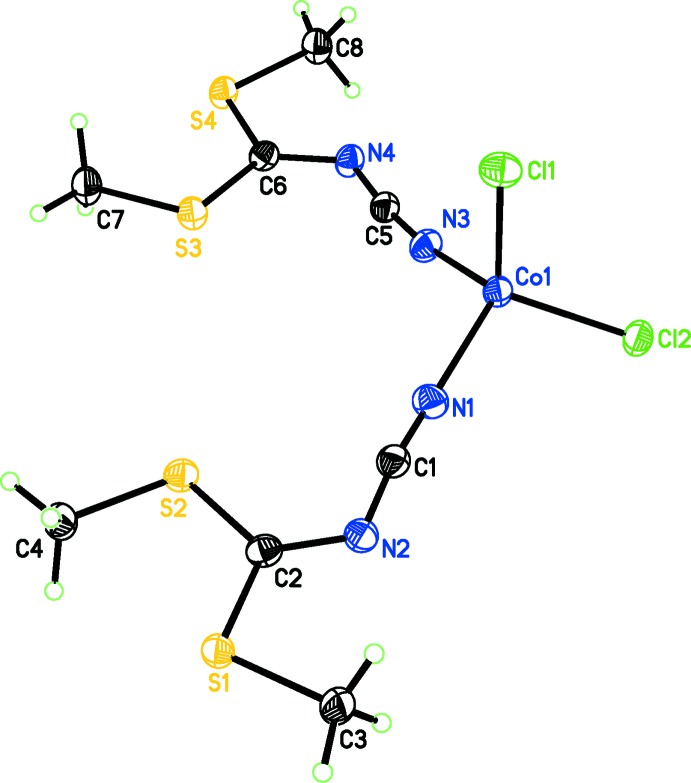
The mol­ecular structure of the title compound, with displacement ellipsoids for non-H atoms drawn at the 50% probability level.

The structure of the title complex consists of a Co^II^ atom coordinated in a distorted tetra­hedral manner by two Cl^−^ ligands and the cyanide N atoms of two dimethyl *N*-cyano­dithio­imino­carbonate ligands (Fig. 1 ▸). Co—Cl and Co—N bond lengths are within expected ranges (Table 1 ▸). The Cl—Co—Cl angle is slightly larger than an ideal tetra­hedral angle whereas three of the Cl—Co—N angles are smaller and the N—Co—N angle is very close to the ideal tetra­hedral angle. This is remarkable because the bulky *N*-cyano­dithio­imino­carbonate ligands might be expected to enforce a higher distortion. The coordination of the ligand’s nitrile nitro­gen atoms to Co^II^ is slightly bent (Table 1 ▸). Despite this bending, the nitrile groups retain triple-bond character, with C1 N1 and C5 N3 bond lengths of 1.148 (3) and 1.147 (3) Å, respectively. The angular sums of the central C atoms of the ligands (C1, C5, 360.0 and 359.98°, respectively) show the expected trigonal–planar configuration. The least-squares planes of the two dimethyl *N*-cyano­dithio­imino­carbonate ligands are almost co-planar [dihedral angle = 5.99 (6)°]. The Co^II^ atom lies 0.437 (2) and 0.557 (2) Å from the mean planes of the two ligands.

## Supra­molecular features   

**Figure 2:**
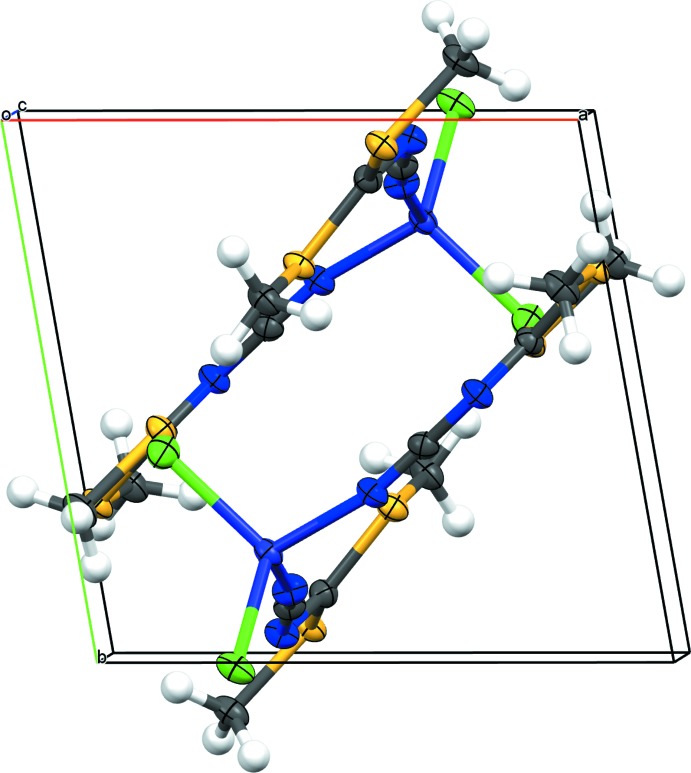
Packing diagram of the title compound viewed approximately along the *c* axis. Displacement ellipsoids are as in Fig. 1 ▸.

The crystal packing features inversion-related pairs of complex mol­ecules (Fig. 2 ▸). These pairs are arranged such that Cl1 is oriented between the H_3_C—S groups of the adjacent mol­ecule, presumably reducing steric inter­actions. Apart from C—H⋯Cl and C—H⋯S inter­actions (Table 2 ▸), π–π stacking with a centroid-to-centroid distance of 3.515 **(su?)** Å prevails within a pair of complex mol­ecules. In the crystal, these pairs are arranged parallel to (110) (Fig. 2 ▸). Additional C—H⋯Cl and C—H⋯S inter­actions between individual pairs consolidate the crystal packing which might be influenced also by other weak contacts under 3.6 Å involving the Cl and S atoms (Table 3 ▸).

## Synthesis and crystallization   

All chemicals were purchased from Aldrich (Germany) and were used as received. The title compound was prepared by mixing of CoCl_2_·6H_2_O (1.665 g, 7 mmol) in aceto­nitrile (30 ml) and dimethyl *N*-cyano­dithio­imino­carbonate (1.023 g, 7 mmol) in aceto­nitrile (20 ml) at room temperature. The resulting blue solution was stirred for about 2 h. Blue crystals suitable for single-crystal X-ray diffraction were obtained after six days of slow solvent evaporation at room temperature (300 K).

Infra-red bands: *ν*(C N) 2224 cm^−1^, *ν*(C=N) 1458 cm^−1^, *ν*(CS_2_) + rocking CH_3_ 1024 and 962 cm^−1^. Melting point 398 K. Elemental analyses of C_8_H_12_Cl_2_CoN_4_S_4_: calculated (found): C 22.75 (21.91), H 2.86 (3.43), N 13.27 (12.63), S 30.37 (29.40).

## Refinement   

Crystal data, data collection and structure refinement details are summarized in Table 4 ▸. Methyl H atoms were allowed to rotate to maximize their contribution to the electron density and were modelled with C—H = 0.98 Å and *U*_iso_(H) = 1.5*U*_eq_(C).

## Supplementary Material

Crystal structure: contains datablock(s) I. DOI: 10.1107/S2056989015023439/wm5249sup1.cif

Structure factors: contains datablock(s) I. DOI: 10.1107/S2056989015023439/wm5249Isup2.hkl

CCDC reference: 1440755

Additional supporting information:  crystallographic information; 3D view; checkCIF report

## Figures and Tables

**Table 1:** Selected geometric parameters (Å, °)

Co1—N3	1.9788 (18)	Co1—Cl2	2.2159 (6)
Co1—N1	1.9791 (19)	Co1—Cl1	2.2291 (6)
			
N3—Co1—N1	110.03 (8)	N1—Co1—Cl1	106.68 (6)
N3—Co1—Cl2	110.81 (6)	Cl2—Co1—Cl1	114.28 (2)
N1—Co1—Cl2	108.76 (6)	C1—N1—Co1	169.31 (18)
N3—Co1—Cl1	106.16 (6)	C5—N3—Co1	167.94 (18)

**Table 2:** Hydrogen-bond geometry (Å, °)

*D*—H⋯*A*	*D*—H	H⋯*A*	*D*⋯*A*	*D*—H⋯*A*
C3—H3*A*⋯Cl2^i^	0.98	2.88	3.538 (2)	125
C4—H4*A*⋯Cl1^ii^	0.98	2.85	3.602 (2)	135
C4—H4*C*⋯Cl2^iii^	0.98	2.74	3.714 (2)	177
C7—H7*A*⋯Cl1^ii^	0.98	2.80	3.592 (3)	138
C7—H7*B*⋯Cl1^iv^	0.98	2.87	3.590 (3)	131
C8—H8*A*⋯Cl2^v^	0.98	2.73	3.450 (2)	131
C8—H8*B*⋯S4^vi^	0.98	2.95	3.910 (2)	167
C8—H8*C*⋯S1^ii^	0.98	2.99	3.709 (3)	131

Symmetry codes: (i) 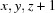; (ii) 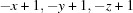; (iii) 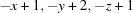; (iv) 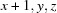; (v) 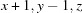; (vi) 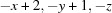.

**Table 3:** Inter­molecular contacts under 3.6 Å

Atom 1⋯Atom 2	Distance (Å)
Cl2⋯S1^i^	3.3742 (11)
Cl2⋯S4^ii^	3.3814 (10)
Cl1⋯S2^iii^	3.5945 (10)

Symmetry codes: (i) *x*, *y*, *z* − 1; (ii) *x* − 1, *y* + 1, *z*; (iii) −*x* + 1, −*y* + 1, −*z* + 1.

**Table 4:** Experimental details

Crystal data	
Chemical formula	[CoCl_2_(C_4_H_6_N_2_S_2_)_2_]
*M*_r_	422.29
Crystal system, space group	Triclinic, *P* 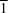
Temperature (K)	120
*a*, *b*, *c* (Å)	8.8533 (10), 8.8722 (10), 11.2487 (14)
α, β, γ (°)	72.823 (3), 87.281 (4), 80.072 (3)
*V* (Å^3^)	831.51 (17)
*Z*	2
Radiation type	Mo *K*α
μ (mm^−1^)	1.85
Crystal size (mm)	0.17 × 0.15 × 0.10

Data collection	
Diffractometer	Bruker Kappa X8 APEXII
Absorption correction	Numerical (*SADABS*; Krause *et al.*, 2015 ▸)
*T*_min_, *T*_max_	0.757, 0.963
No. of measured, independent and observed [*I* > 2σ(*I*)] reflections	12851, 4139, 3566
*R*_int_	0.028
(sin θ/λ)_max_ (Å^−1^)	0.669

Refinement	
*R*[*F* ^2^ > 2σ(*F* ^2^)], *wR*(*F* ^2^), *S*	0.033, 0.087, 1.09
No. of reflections	4139
No. of parameters	176
H-atom treatment	H-atom parameters constrained
Δρ_max_, Δρ_min_ (e Å^−3^)	1.11, −0.50

Computer programs: *APEX2* and *SAINT* (Bruker, 2014 ▸), *SHELXT2014* (Sheldrick, 2015*a* ▸), *SHELXL2014* (Sheldrick, 2015*b* ▸), *XP* in *SHELXTL* (Sheldrick, 2008 ▸), *Mercury* (Macrae *et al.*, 2006 ▸) and *publCIF* (Westrip, 2010 ▸).

## supplementary crystallographic information

### Crystal data

**Table:**

[CoCl_2_(C_4_H_6_N_2_S_2_)_2_]	*Z* = 2
*M**_r_* = 422.29	*F*(000) = 426
Triclinic, *P*1	*D*_x_ = 1.687 Mg m^−^^3^
*a* = 8.8533 (10) Å	Mo *K*α radiation, λ = 0.71073 Å
*b* = 8.8722 (10) Å	Cell parameters from 6476 reflections
*c* = 11.2487 (14) Å	θ = 2.3–28.4°
α = 72.823 (3)°	µ = 1.85 mm^−^^1^
β = 87.281 (4)°	*T* = 120 K
γ = 80.072 (3)°	Block, blue
*V* = 831.51 (17) Å^3^	0.17 × 0.15 × 0.10 mm

### Data collection

**Table:**

Bruker Kappa X8 APEXII diffractometer	4139 independent reflections
Radiation source: fine-focus sealed tube	3566 reflections with *I* > 2σ(*I*)
Graphite monochromator	*R*_int_ = 0.028
Detector resolution: 8.33 pixels mm^-1^	θ_max_ = 28.4°, θ_min_ = 1.9°
combination of ω and φ–scans	*h* = −11→11
Absorption correction: numerical (*SADABS*; Krause *et al.*, 2015)	*k* = −11→11
*T*_min_ = 0.757, *T*_max_ = 0.963	*l* = −14→7
12851 measured reflections	

### Refinement

**Table:**

Refinement on *F*^2^	Primary atom site location: real-space vector search
Least-squares matrix: full	Secondary atom site location: difference Fourier map
*R*[*F*^2^ > 2σ(*F*^2^)] = 0.033	Hydrogen site location: inferred from neighbouring sites
*wR*(*F*^2^) = 0.087	H-atom parameters constrained
*S* = 1.09	*w* = 1/[σ^2^(*F*_o_^2^) + (0.0454*P*)^2^ + 0.4168*P*] where *P* = (*F*_o_^2^ + 2*F*_c_^2^)/3
4139 reflections	(Δ/σ)_max_ = 0.001
176 parameters	Δρ_max_ = 1.11 e Å^−^^3^
0 restraints	Δρ_min_ = −0.50 e Å^−^^3^

### Special details

**Table:**

Geometry. All e.s.d.'s (except the e.s.d. in the dihedral angle between two l.s. planes) are estimated using the full covariance matrix. The cell e.s.d.'s are taken into account individually in the estimation of e.s.d.'s in distances, angles and torsion angles; correlations between e.s.d.'s in cell parameters are only used when they are defined by crystal symmetry. An approximate (isotropic) treatment of cell e.s.d.'s is used for estimating e.s.d.'s involving l.s. planes.

### Fractional atomic coordinates and isotropic or equivalent isotropic displacement parameters (Å^2^)

**Table:**

	*x*	*y*	*z*	*U*_iso_*/*U*_eq_	
Co1	0.32319 (3)	0.80109 (3)	0.23799 (3)	0.01930 (9)	
Cl1	0.16996 (6)	0.61729 (6)	0.29242 (6)	0.02787 (13)	
Cl2	0.23586 (6)	1.01243 (6)	0.08085 (5)	0.02677 (13)	
S1	0.35461 (6)	0.94916 (6)	0.80748 (5)	0.02145 (12)	
S2	0.53818 (6)	0.72624 (6)	0.68536 (5)	0.02235 (12)	
S3	0.83867 (6)	0.41595 (6)	0.37512 (5)	0.02199 (12)	
S4	0.97640 (6)	0.27842 (6)	0.17733 (5)	0.02215 (12)	
N1	0.3446 (2)	0.8728 (2)	0.38617 (18)	0.0247 (4)	
N2	0.3115 (2)	0.9569 (2)	0.57616 (17)	0.0223 (4)	
N3	0.5233 (2)	0.6904 (2)	0.19638 (19)	0.0242 (4)	
N4	0.73672 (19)	0.5076 (2)	0.14206 (18)	0.0220 (4)	
C1	0.3361 (2)	0.9056 (2)	0.4779 (2)	0.0217 (4)	
C2	0.3924 (2)	0.8854 (2)	0.6783 (2)	0.0206 (4)	
C3	0.1932 (3)	1.1047 (3)	0.7548 (2)	0.0266 (5)	
H3A	0.1614	1.1553	0.8205	0.040*	
H3B	0.1081	1.0588	0.7346	0.040*	
H3C	0.2217	1.1849	0.6804	0.040*	
C4	0.6032 (2)	0.6665 (3)	0.8441 (2)	0.0261 (4)	
H4A	0.6846	0.5730	0.8579	0.039*	
H4B	0.5173	0.6391	0.9001	0.039*	
H4C	0.6429	0.7547	0.8608	0.039*	
C5	0.6267 (2)	0.6032 (2)	0.1776 (2)	0.0208 (4)	
C6	0.8401 (2)	0.4120 (2)	0.2233 (2)	0.0201 (4)	
C7	1.0010 (3)	0.2699 (3)	0.4438 (2)	0.0288 (5)	
H7A	1.0052	0.2589	0.5330	0.043*	
H7B	1.0951	0.3042	0.4038	0.043*	
H7C	0.9915	0.1667	0.4323	0.043*	
C8	0.9185 (3)	0.3104 (3)	0.0198 (2)	0.0271 (5)	
H8A	0.9860	0.2358	−0.0163	0.041*	
H8B	0.9255	0.4205	−0.0290	0.041*	
H8C	0.8125	0.2924	0.0188	0.041*	

### Atomic displacement parameters (Å^2^)

**Table:**

	*U*^11^	*U*^22^	*U*^33^	*U*^12^	*U*^13^	*U*^23^
Co1	0.01959 (14)	0.02016 (14)	0.01816 (16)	0.00228 (10)	−0.00075 (11)	−0.00863 (11)
Cl1	0.0279 (3)	0.0291 (3)	0.0300 (3)	−0.0061 (2)	0.0048 (2)	−0.0138 (2)
Cl2	0.0319 (3)	0.0244 (2)	0.0215 (3)	0.00654 (19)	−0.0057 (2)	−0.0084 (2)
S1	0.0240 (2)	0.0225 (2)	0.0175 (3)	0.00254 (19)	−0.00157 (19)	−0.0087 (2)
S2	0.0242 (2)	0.0216 (2)	0.0215 (3)	0.00096 (19)	0.0012 (2)	−0.0095 (2)
S3	0.0212 (2)	0.0245 (2)	0.0197 (3)	0.00097 (18)	0.00047 (19)	−0.0084 (2)
S4	0.0208 (2)	0.0231 (2)	0.0210 (3)	0.00428 (18)	0.00045 (19)	−0.0086 (2)
N1	0.0309 (9)	0.0249 (9)	0.0192 (9)	−0.0058 (7)	0.0009 (7)	−0.0072 (7)
N2	0.0273 (9)	0.0222 (8)	0.0175 (9)	−0.0008 (7)	−0.0002 (7)	−0.0076 (7)
N3	0.0236 (8)	0.0217 (8)	0.0266 (10)	0.0018 (7)	−0.0005 (7)	−0.0088 (7)
N4	0.0210 (8)	0.0222 (8)	0.0211 (9)	0.0025 (6)	0.0004 (7)	−0.0070 (7)
C1	0.0249 (10)	0.0194 (9)	0.0202 (11)	−0.0039 (7)	−0.0002 (8)	−0.0047 (8)
C2	0.0214 (9)	0.0202 (9)	0.0208 (11)	−0.0032 (7)	0.0032 (8)	−0.0077 (8)
C3	0.0295 (11)	0.0250 (10)	0.0239 (11)	0.0063 (8)	−0.0046 (9)	−0.0105 (9)
C4	0.0246 (10)	0.0272 (10)	0.0259 (12)	0.0043 (8)	−0.0055 (9)	−0.0109 (9)
C5	0.0231 (9)	0.0195 (9)	0.0190 (10)	−0.0020 (7)	−0.0014 (8)	−0.0049 (8)
C6	0.0198 (9)	0.0197 (9)	0.0203 (10)	−0.0013 (7)	0.0020 (8)	−0.0067 (8)
C7	0.0282 (11)	0.0317 (11)	0.0235 (12)	0.0036 (9)	−0.0040 (9)	−0.0077 (9)
C8	0.0295 (11)	0.0309 (11)	0.0199 (11)	0.0046 (9)	−0.0007 (9)	−0.0113 (9)

### Geometric parameters (Å, º)

**Table:**

Co1—N3	1.9788 (18)	N3—C5	1.147 (3)
Co1—N1	1.9791 (19)	N4—C5	1.306 (3)
Co1—Cl2	2.2159 (6)	N4—C6	1.321 (3)
Co1—Cl1	2.2291 (6)	C3—H3A	0.9800
S1—C2	1.708 (2)	C3—H3B	0.9800
S1—C3	1.793 (2)	C3—H3C	0.9800
S2—C2	1.726 (2)	C4—H4A	0.9800
S2—C4	1.798 (2)	C4—H4B	0.9800
S3—C6	1.718 (2)	C4—H4C	0.9800
S3—C7	1.793 (2)	C7—H7A	0.9800
S4—C6	1.714 (2)	C7—H7B	0.9800
S4—C8	1.795 (2)	C7—H7C	0.9800
N1—C1	1.148 (3)	C8—H8A	0.9800
N2—C1	1.310 (3)	C8—H8B	0.9800
N2—C2	1.314 (3)	C8—H8C	0.9800
			
N3—Co1—N1	110.03 (8)	H3B—C3—H3C	109.5
N3—Co1—Cl2	110.81 (6)	S2—C4—H4A	109.5
N1—Co1—Cl2	108.76 (6)	S2—C4—H4B	109.5
N3—Co1—Cl1	106.16 (6)	H4A—C4—H4B	109.5
N1—Co1—Cl1	106.68 (6)	S2—C4—H4C	109.5
Cl2—Co1—Cl1	114.28 (2)	H4A—C4—H4C	109.5
C2—S1—C3	100.97 (10)	H4B—C4—H4C	109.5
C2—S2—C4	103.59 (10)	N3—C5—N4	172.7 (2)
C6—S3—C7	103.88 (10)	N4—C6—S4	119.43 (16)
C6—S4—C8	101.50 (10)	N4—C6—S3	121.39 (15)
C1—N1—Co1	169.31 (18)	S4—C6—S3	119.16 (12)
C1—N2—C2	120.71 (18)	S3—C7—H7A	109.5
C5—N3—Co1	167.94 (18)	S3—C7—H7B	109.5
C5—N4—C6	120.10 (19)	H7A—C7—H7B	109.5
N1—C1—N2	172.7 (2)	S3—C7—H7C	109.5
N2—C2—S1	120.19 (15)	H7A—C7—H7C	109.5
N2—C2—S2	121.18 (16)	H7B—C7—H7C	109.5
S1—C2—S2	118.63 (13)	S4—C8—H8A	109.5
S1—C3—H3A	109.5	S4—C8—H8B	109.5
S1—C3—H3B	109.5	H8A—C8—H8B	109.5
H3A—C3—H3B	109.5	S4—C8—H8C	109.5
S1—C3—H3C	109.5	H8A—C8—H8C	109.5
H3A—C3—H3C	109.5	H8B—C8—H8C	109.5
			
C1—N2—C2—S1	−177.67 (16)	C5—N4—C6—S4	175.87 (16)
C1—N2—C2—S2	2.3 (3)	C5—N4—C6—S3	−2.8 (3)
C3—S1—C2—N2	2.6 (2)	C8—S4—C6—N4	−3.5 (2)
C3—S1—C2—S2	−177.38 (13)	C8—S4—C6—S3	175.25 (13)
C4—S2—C2—N2	−176.86 (18)	C7—S3—C6—N4	−178.88 (18)
C4—S2—C2—S1	3.11 (15)	C7—S3—C6—S4	2.43 (16)

### Hydrogen-bond geometry (Å, º)

**Table:**

*D*—H···*A*	*D*—H	H···*A*	*D*···*A*	*D*—H···*A*
C3—H3*A*···Cl2^i^	0.98	2.88	3.538 (2)	125
C4—H4*A*···Cl1^ii^	0.98	2.85	3.602 (2)	135
C4—H4*C*···Cl2^iii^	0.98	2.74	3.714 (2)	177
C7—H7*A*···Cl1^ii^	0.98	2.80	3.592 (3)	138
C7—H7*B*···Cl1^iv^	0.98	2.87	3.590 (3)	131
C8—H8*A*···Cl2^v^	0.98	2.73	3.450 (2)	131
C8—H8*B*···S4^vi^	0.98	2.95	3.910 (2)	167
C8—H8*C*···S1^ii^	0.98	2.99	3.709 (3)	131

Symmetry codes: (i) *x*, *y*, *z*+1; (ii) −*x*+1, −*y*+1, −*z*+1; (iii) −*x*+1, −*y*+2, −*z*+1; (iv) *x*+1, *y*, *z*; (v) *x*+1, *y*−1, *z*; (vi) −*x*+2, −*y*+1, −*z*.
